# *Leishmania* spp. Epidemiology of Canine Leishmaniasis in the Yucatan Peninsula

**DOI:** 10.1100/2012/945871

**Published:** 2012-05-09

**Authors:** A. López-Céspedes, S. S. Longoni, C. H. Sauri-Arceo, M. Sánchez-Moreno, R. I. Rodríguez-Vivas, F. J. Escobedo-Ortegón, M. A. Barrera-Pérez, M. E. Bolio-González, C. Marín

**Affiliations:** ^1^Departamento de Parasitología, Facultad de Ciencias, Universidad de Granada, Severo Ochoa s/n, 18071-Granada, Spain; ^2^Facultad de Medicina Veterinaria y Zootecnia, Campus de Ciencias Biológicas y Agropecuarias, Universidad Autónoma de Yucatán, Km 15.5 Carretera Mérida-Xmatkuil, 4-116 Itzimná, Mérida, Yucatan, Mexico; ^3^Laboratorio de Zoonosis y VBD's, C.I.R. Dr. Hideyo Noguchi, Universidad Autónoma de Yucatán, Avenida Itzaes No. 490 × 59, 97000 Mérida, Yucatan, Mexico

## Abstract

Canine Leishmaniasis is widespread in various Mexican states, where different species of *Leishmania* have been isolated from dogs. In the present study, we describe the detection of *L. braziliensis, L. infantum,* and *L. mexicana* in serum of dogs from the states of Yucatan and Quintana Roo in the Yucatan Peninsula (Mexico). A total of 412 sera were analyzed by ELISA using the total extract of the parasite and the iron superoxide dismutase excreted by different trypanosomatids as antigens. We found the prevalence of *L. braziliensis *to be 7.52%, *L. infantum* to be 6.07%, and *L. mexicana* to be 20.63%, in the dog population studied. The results obtained with ELISA using iron superoxide dismutase as the antigen were confirmed by western blot analysis with its greater sensitivity, and the agreement between the two techniques was very high.

## 1. Introduction

Leishmaniasis is caused by a protozoan parasite called *Leishmania*, of which 21 species have been identified as pathogenic to humans. In most cases, it causes disease in animals and humans that become infected by accidentally entering endemic areas. It is a cosmopolitan disease of the most complex and diverse type, with significant overlap between different *Leishmania* species and their vectors, determining a complex ecology and epidemiology. It has three clinical forms: cutaneous, mucocutaneous, and visceral [1], with the visceral form being the most severe.

The disease is one of the less understood diseases of the world, affecting mainly developing countries. It is believed that about 350 million people are at risk of contracting the disease and more than 2 million new infections are recorded each year. Control programs of Leishmaniasis remain weak, showing a worrying increase in both mortality and morbidity in the world [2].

Dogs infected with this protozoan are the main reservoir of the disease and play a key role in its transmission to humans. Growing awareness that the control in humans depends on effective control of canine leishmaniasis has been promoted in recent years. Research on *Leishmania* infection in dogs has been conducted with hope of not only reducing the burden of disease in dogs, but also reducing the incidence of human leishmaniasis [3].

Canine Leishmaniasis is widespread in South America and is among the more important canine vector-borne diseases that occur in the region, mainly because of its great zoonotic relevance. Thus, many species of *Leishmania* have been isolated and molecularly characterized in dogs in South America, including *L. amazonensis*, *L. braziliensis*, *L. colombiensis*, *L. infantum* (syn. *L. chagasi*), *L. mexicana*, *L. panamensis*, *L. peruviana*, and *L. pifanoi* [4, 5]. *L. infantum* is the causative agent of canine visceral leishmaniasis, the most important form in South America, where dogs are its main reservoir [6]. *L. braziliensis* is the main causative agent of cutaneous leishmaniasis in dogs in this region [7].

The prevalence of *Leishmania* spp. in dogs in South America varies widely between regions. Depending on the diagnostic method used, the prevalence is usually from 25%, to a maximum of 75% in endemic foci [8]. It is difficult to estimate the overall prevalence of *Leishmania* infection in dogs in South America due to the limited number of publications in some countries, the existence of methodological differences between studies (sample size or positivity criterion) and the limitations of serology (such as cross-reactions).

 An important epidemiological feature is that dogs infected with *L. infantum* are apparently healthy, showing no clinical signs evident of the disease. In studies carried out in Brazil, 80% of seropositive dogs showed no symptoms of the disease. This information is critical because seropositive, but apparently healthy dogs, are a source of infection for flebotomos [9, 10].

Due to the variety of clinical signs of canine leishmaniasis, the diagnosis is very difficult. Several methods have therefore been developed to facilitate this task. However, it is essential to understand the basis of each test, its limitations and its clinical interpretation, and to consider the combination of more than one diagnostic test [11].

Many studies have been performed to discover a *Leishmania*-specific antigen that could increase the specificity of serodiagnosis [12–14]. One such candidate antigen is excreted iron-superoxide dismutase (Fe-SODe). This was found to be highly immunogenic and specific and is, therefore, a useful molecular marker for diagnosing infection with these parasites. The antigen was shown to provide good results in the diagnosis of visceral canine leishmaniasis [15] and of both cutaneous and mucocutaneous leishmaniasis in human serum in Peru [16]. It was also used in the diagnosis of visceral, cutaneous, and mucocutaneous leishmaniasis in dog serum in Mexico [17].

Here, we present the development of an ELISA using the antigen Fe-SODe excreted by *L. mexicana*, *L. braziliensis*, and *L. infantum* as a basis for the development of a sero-diagnostic tool. Further, as a test of this method, we describe the percentage of stray dogs infected with these parasites at Molas, Xmatkuil, Playa del Carmen, Akumal, Xcalac and Xahuaxol on the Yucatan Peninsula (Mexico). We also demonstrate the lack of cross-reactions between the different *Leishmania* spp. and other trypanosomatids such as *T. cruzi*. The use of Fe-SODe could have significant diagnostic and epidemiological value for both canine leishmaniasis and other parasitic diseases caused by different species of the genus *Leishmania*.

## 2. Materials and Methods

### 2.1. Parasites and Culture

Promastigotes of *L.* *braziliensis* (MHOM/BR/75/M2904), *L.* *infantum* (MCAN/ES/2001/UCM-10), *L.* *mexicana* (MHOM/BZ/82/Bel 21), were grown in axenic medium trypanosomes liquid (MTL; Gibco) supplemented with 10% heat-inactivated fetal bovine serum at 28°C in Falcon flasks.

### 2.2. Area of Study

**Figure 1:**
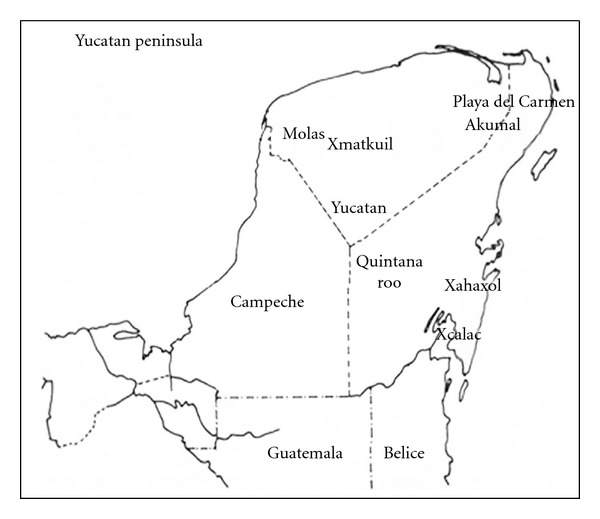
Map of theYucatan Peninsula,Mexico, showing the geographical location of Molas and Xmatkuil in Yucatan State and Playa del Carmen, Akumal, Xcalac and Xahuachol in Quintana Roo State.

The study was made in the towns of Molas and Xmatkuil in the state of Yucatan, and in the towns of Playa del Carmen, Akumal, Xcalac, and Xahaxol state of Quintana Roo (Figure 1). Both states have tropical and subtropical climates with warm and wet conditions, with annual average temperatures of 28°C (the maximum being 40°C) and average humidity of 72%, with annual rainfall of 1100 mm.

### 2.3. Dog Populations and Collection of Samples

Of a total of 412 sera studied, 173 were collected in the state of Yucatan, with 147 in the region of the town Molas and 26 in Xmatkuil. The other 239 sera were collected in the state of Quintana Roo, with 63 in Playa del Carmen, 36 in Akumal, 127 in Xcalac, and 13 in Xahuaxol. For simplicity in presenting the results, the total of 412 sera was segregated into the following: sera 1–147 from Molas; 148–210 from Playa del Carmen; 211–246 from Akumal; 247–272 from Xmatkuil; 273–399 from Xcalac; 400–412 from Xahuaxol.

A sample of 5 mL of whole blood was drawn from the cephalic vein of each dog into assay tubes (Vacuttainer, Beckton-Dickinson, USA) and kept at 4°C. The negative control sera (from 20 healthy or asymptomatic dogs) were taken from stray dogs put down by the veterinary services in Granada (Spain), which were not reactive to the Western-blot techniques.

### 2.4. Whole-Parasite Extract (Fraction H)

The parasite culture (in the exponential growth phase) was concentrated by centrifugation at 1500 rpm for 10 min. The cell pellet was then washed twice with phosphate-buffered saline (PBS) and resuspended in ice-cold sodium Tris/HCl ethylenediamine tetraacetic acid (EDTA) buffer (0.25 M sucrose, 25 mM Tris/HCl, 1 mM EDTA, pH 7.8; buffer 1). The resulting pellet (0.5–0.6 g wet weight mL^−1^) was suspended in 3 mL of buffer 1 and disrupted by three cycles of sonic disintegration (30 s each at 60 V). The sonicated homogenate was centrifuged at 1500 rpm for 10 min at 4°C, and then the pellet was washed three times with buffer 1 for a total supernatant fraction of 9 mL. This fraction was centrifuged (2500 rpm for 10 min at 4°C) and the supernatant (fraction H) was collected.

### 2.5. Extraction and Partial Purification of the SOD Excreted (Fe-SODe)

Parasite forms in the exponential growth phase, obtained as described earlier, were concentrated by centrifugation at 1500 rpm for 10 min, the cell pellet was washed twice in serum free MTL medium, then the number of cells was counted in an hemocytometric chamber, and the cells were distributed into aliquots of 5 × 10^9^ parasites/mL. Subsequently, the parasites were again grown in serum-free MTL medium for 24 h, the supernatant was collected by centrifugation at 1500 rpm for 10 min and then passed through a filter of 0.45-*μ*m pore size, and solid ammonium sulfate was added. The protein fraction, which precipitated at a salt concentration of between 35% and 85%, was centrifuged (9000 rpm for 20 min at 4°C), redissolved in 2.5 mL of 20 mM potassium phosphate buffer (pH 7.8) containing 1 mM EDTA (buffer 2), and dialyzed on a Sephadex G-25 column (Pharmacia, PD 10) previously balanced with buffer 2, to give a final volume of 2.5 mL (Fe-SODe fraction) [18].

The H and Fe-SODe fractions were both used as antigen fractions in ELISA and Western blot assays. The protein content was determined using the Bradford reagent, based on the Bradford method (Sigma Immunochemical), with bovine serum albumin as standard [15].

### 2.6. Serological Assay: ELISA

Fractions H and Fe-SODe from the parasites (*L. braziliensis*, *L. infantum,* and *L. mexicana*), cultured and processed as described earlier, were used as the antigen fraction for the ELISA assay in all cases. The total homogenate (fraction H) and semi-purified protein fraction (Fe-SODe), at a concentration of 5 and 1.5 *μ*g, respectively, were coated onto polystyrene microtiter plates (Nunc) in carbonate buffer (pH 8.2) for 2 h at 37°C. The antigen remaining on the plate was eliminated by washing three times with PBS-Tween 20 0.05% (washing buffer). Free adsorption sites were blocked by incubation (2 h at 37°C) with blocking buffer (PBS-Tween 20 0.2%, bovine serum albumin 1%). The antibodies retained at a serum dilution of 1/100 in PBS were developed with peroxidase-labeled sheep anti-total-dog immunoglobulin antibodies at a dilution of 1 : 1000 as conjugate. The enzyme reaction was developed in the dark with the chromogenic substrate o-phenylenediamine dihydrochloride (Sigma, Madrid, Spain) and 10 *μ*L of 30% H_2_O_2_ per 25 mL for 20 min. The reaction was stopped by addition of 50 *μ*L of 3 N HCl, and the absorbance was read at 492 nm in a microplate reader (Metertech Σ 960). All samples were analyzed in triplicate in polystyrene microtiter plates, and the mean and standard deviations of the optical densities of the negative control sera (10 healthy dogs) were used to calculate the cutoff value (mean + 3 standard deviation).

### 2.7. Western Blot Analysis

The antigen fraction of Fe-SODe (at a concentration of 1.5 *μ*g protein) from *L. braziliensis*, *L. mexicana.,* and *L. infantum* was run on Isoelectric focusing (IEF) 3–9 gels and then transferred to nitrocellulose, for 30 min, as described in the Phast System manual. The membrane was blocked for 2 h at room temperature using 0.4% gelatin and 0.2% Tween 20 in PBS, then washed three times with 0.1% Tween 20 in PBS (PBS-T), and incubated for 2 h at room temperature with dog sera at a dilution of 1/100. Before washing again, the membrane was further incubated for 2 h at room temperature with a second antibody, namely anti-dog immunoglobulin G (Fc-specific) peroxidase conjugate (Sigma Immunochemical; dilution 1/1000). After washing as described earlier, the substrate diaminobenzidine (0.5 mg/mL in buffer Tris/HCl 0.1 M, pH 7.4, containing 1/5000 H_2_O_2_ [10 v/v]) was added and the reaction was stopped by washing several times with distilled water.

## 3. Results

The 412 sera of dogs from different regions of the Yucatan Peninsula (173 from the state of Yucatan and 239 from the state of Quintana Roo) were assayed by ELISA using the total extract of the parasite (H) and the excreted iron-superoxide dismutase (Fe-SODe) of *L. braziliensis*, *L. infantum* and *L. mexicana* as the antigen.

The results obtained with the ELISA using the total extract as antigen (ELISA/H) indicated the prevalence of *L. braziliensis* to be 2.18% in the peninsula. In the state of Yucatan, four positive sera were detected (prevalence = 2.31%) of which three were from Xmatkuil (prevalence = 11.54%) and one was from Molas (prevalence = 0.68%). In the state of Quintana Roo, there were five positive sera (prevalence = 2.09%), one from Playa del Carmen (prevalence = 1.59%), another from Akumal (prevalence = 2.78%), and three from Xcalac (prevalence = 2.36%). As for the ELISA/Fe-SODe, the prevalence of *L. braziliensis* detected from total sera from dogs analyzed was 7.52%, with 11.56% in the state of Yucatan and 4.60% in the state of Quintana Roo (Table 1). Fifteen sera were detected as positive from Molas (prevalence = 10.20%), five were positive from Xmatkuil (prevalence = 19.23%), two from Playa del Carmen (prevalence = 3.17%), three from Akumal (prevalence = 8.33%), and six from Xcalac (prevalence = 4.72%) (Table 2). To verify these data, sera were analyzed by western blot analysis using Fe-SODe as the antigen (western blot analysis/Fe-SODe). This showed the prevalence of *L. braziliensis* to be 6.31% in the peninsula, 8.67% in the state of Yucatan, and 4.60% in Quintana Roo. The agreement between the ELISA/Fe-SODe and western blot analysis/Fe-SODe for *L. braziliensis* was 84%.

The prevalence of *L. infantum* indicated by ELISA/H was 0.49%, with only two positive sera; one serum from Molas (prevalence = 0.68%) and one from Xcalac (prevalence = 0.79%). However, the prevalence of total sera tested with ELISA/Fe-SODe was 6.07%, with three positive sera from Molas (prevalence = 2.04%), twenty positive sera from Xcalac (prevalence = 15.75%), and two positive sera from Playa del Carmen (prevalence = 3.17%, Table 3). Using western blot analysis/Fe-SODe of *L. infantum,* the total prevalence was 5.58%, with 2.04% in Molas, 14.17% in Xcalac and 3.17% in Playa del Carmen. The agreement between the techniques (ELISA/Fe-SODe and western blot analysis/Fe-SODe) for *L. infantum* was 92%.

The ELISA/H for *L. mexicana* resulted in 16 positive sera from the total (prevalence = 3.88%). Eight positive sera were from the state of Yucatan with seven from Molas (prevalence = 4.78%) and one from Xmatkuil (prevalence = 3.85%). The other eight positive sera were from the state of Quintana Roo; two from Playa del Carmen (prevalence = 3.17%), three from Akumal (prevalence = 8.33%), two from Xcalac (prevalence = 1.57%), and one from Xahaxol (prevalence = 7.69%). However, the ELISA/Fe-SODe resulted in the detection of 85 positive sera from the total indicating a prevalence of 20.63% (Table 1). From these, 46 positive sera were from the state of Yucatan (prevalence = 26.59%), with 45 positive from Molas (prevalence = 30.61%) and one from Xmatkuil (prevalence = 3.85%).Thirty-nine positive sera were from Quintana Roo (prevalence = 16.32%) with fifteen from Playa del Carmen (prevalence = 23.81%), eleven from Akumal (prevalence = 30.56%), twelve from Xcalac (prevalence = 9.45%), and one from Xahuaxol (prevalence = 7.69%, Table 4). These data were also tested by western blot analysis obtaining 84 positive sera from the total, indicating a prevalence of 20.39%, with 45 positive sera from the state of Yucatan (prevalence = 26.01%) and 39 from Quintana Roo (prevalence = 16.32%). The agreement between the ELISA/Fe-SODe and western blot analysis/Fe-SODe for *L. mexicana* was 99%.

The use of Fe-SODe as the antigenic fraction is very sensitive for the detection of leishmaniasis, detecting a very small number of false positives [19]. To demonstrate the possible absence of cross-reactivity between the different species of *Leishmania*, ELISA studies were performed with the Fe-SODe of *L. mexicana*, *L. infantum,* and *L. braziliensis* as the antigen fraction to analyze the 412 canine sera. Only four sera (0.97%) were positive for the three species of *Leishmania*. 

However, 17 sera were positive to both *L. mexicana* and *L. braziliensis* indicating a prevalence of both species simultaneously to be 4.13%. With respect to *L. mexicana* and *L. infantum*, only 9 sera were positive for both species (prevalence = 2.18%). Only eight sera were positive for both *L. braziliensis* and *L. infantum*, indicating a prevalence of 1.94% (Table 5).

To verify the data, canine sera were also analyzed by western blot analysis using Fe-SODe of the three species of *Leishmania* as the antigenic fraction. The sensitivity obtained for the ELISA/Fe-SODe over the western blot was 96.2% for *L. braziliensis* and 100% for *L. infantum* and *L. mexicana*. The specificity obtained with this technique was 99% for *L. braziliensis*, 99.7% for *L. mexicana,* and 99.5% for *L. infantum*. The Kappa value was 1 for the three species of *Leishmania*, as seen in Table 6.

## 4. Discussion

In 1993, three dogs were diagnosed with leishmaniasis, whose owners were also infected in the state of Quintana Roo, Mexico [20]. This relationship between dogs and people infected with leishmaniasis was previously observed in other countries such as Argentina and Brazil [21, 22]. 

The increase in the canine population, in addition to the social role of dogs in different cultures, makes the relationship of dogs with humans even closer. The risk of human infection increases with greater relationships between dogs and their wild environment. The risk of human infection also increases with the presence of vectors in the owners homes, which is increasingly common given the successful adaptation of blood-sucking arthropods (mosquitoes, ticks, fleas, flies, etc.) in the domestic environment [23].

Numerous studies support the possibility that dogs are the most important reservoirs for leishmaniasis, cutaneous, mucocutaneous, and visceral, in humans [20, 24, 25]. It is therefore, necessary to control and monitor these animals to prevent them from becoming a problem for human health.

In Mexico, and more specifically in the Yucatan Peninsula, which is an area considered endemic for leishmaniasis, susceptibility of dogs to *Leishmania* resembles that described in other Latin American countries. However, it appears that the dogs studied in this area seem to be exposed to greater risks of infection than their counterparts in other countries studied [20].

In this study, we found that the dominant species in dogs in the Yucatan Peninsula is *L. mexicana*, where we found the prevalence to be 20.63% with the ELISA/Fe-SODe. These data are consistent with those published by other authors [20, 26]. If we analyze the prevalence values according to the origin of the dogs, we found that the prevalence of *L. mexicana* in dogs from the state of Quintana Roo is 16.32%, while the prevalence in dogs from the state of Yucatan is 26.59%.

In 2005, Andrade and colleagues suspected the presence of *L. braziliensis* in the Yucatan Peninsula. Previous studies in dogs living in wild areas in Tulum and Celestum (Yucatan Peninsula) showed a significant presence of this species with the prevalence at 32.8% [17]. In another study on canine sera from the Canine and Feline Control Center and from private clinics in Merida (Yucatan State), the prevalence of *L. braziliensis* was found to be 8.2% [27]. On this occasion, we found the prevalence to be 7.52% in the Yucatan Peninsula, corresponding to 11.56% in the state of Yucatan and 4.60% in Quintana Roo. The presence of *L. braziliensis* in Mexico and, more specifically, in the Yucatan Peninsula, was only recently discovered. The spread of this species from the displacement of the vector and/or reservoirs from the neighboring countries, Belize and Guatemala, where the disease has been reported [2, 28, 29] is now very clear. This may explain the prevalence of 4.72% for *L. braziliensis* detected in Xcalac, a town located near Belize (Figure 1).

The species causing visceral leishmaniasis in the American continent is *L. infantum* (*syn. chagasi*). The role of the vector, *Lutzomyia  longipalpis*, involved in transmission in this area, and the dog's role as a reservoir for this parasite [24], are now understood. Previous studies demonstrated the presence of *L. infantum* (syn. *chagasi*) in the city of Merida (Yucatan), with prevalence of 11.9% [27]. In our study, we obtained a prevalence of 6.07% in the Yucatan Peninsula. There is a very large variation in the prevalence of *L. infantum* (syn. *chagasi*) within the two states sampled. In the state of Yucatan we found, a prevalence of only 1.73%, while in the state of Quintana Roo we found, a prevalence of 9.21%. Of note is the prevalence of *L. infantum* of 15.75% detected in the city of Xcalac (Quintana Roo). This could be due to the proximity between the region in which the city of Xcalac lies and the countries of Belize and Guatemala, where there is a high prevalence of *L. infantum* [2].

In this study, we found that the prevalence of antibodies to *L. mexicana *in the dog sera tested is much higher than those found against *L. braziliensis* and *L. infantum* (*syn. chagasi*). Specific antibodies of different species of *Leishmania* have been detected in more than one dog studied. However, this was not due to cross-reaction as the prevalence of multiantibodies is relatively low and the antibodies are very distinct from each other (Table 5). Therefore, cases of co-infections can be considered.

Western blot analysis has a higher sensitivity than ELISA. When comparing the results obtained from the ELISA/Fe-SODe to those obtained from the western blot analysis, we found very high values of agreement between the two techniques, being 99% for *L. mexicana*, 84% for *L. braziliensis,* and 92% for *L. infantum*. 

However, due to its low cost and easy reproducibility, very important considerations in low socioeconomic countries in which leishmaniasis is endemic, ELISA/Fe-SODe may be viewed as the more ideal technique. It has specificity between 99% and 99.7%, sensitivity between 96.2% and 100%, a positive predictive value between 86.2% and 98.8%, a negative predictive value between 99.7% and 100% and a Kappa index of 1 (Table 6).

In summary, this study demonstrates the presence of at least three species of *Leishmania* (*L. mexicana*, *L. braziliensis,* and *L. infantum*) in the canine populations of the towns of Playa del Carmen, Akumal, Xcalac, Xahuaxol (Quintana Roo State), Molas, and Xmatkuil (Yucatan State). Dogs can therefore be regarded as an important source of transmission of leishmaniasis. A thorough epidemiological study of this population, both wild and urban, to design an effective control plan against this disease is, therefore, required. Such a study could be extended to other Mexican states such as Campeche and other neighboring countries. Because of the close relationship between dogs and their owners, a similar study should be done with the human population in these regions. Finally, these results provide further confirmation that Fe-SODe is a good antigen fraction for serodiagnosis in epidemiological studies.

## Figures and Tables

**Table 1:** Prevalence of antibodies to *Leishmania braziliensis*, *L. infantum* and *L. mexicana* infection in dogs from Peninsula of Yucatan (Mexico).

*Leishmania mexicana*					
Yucatan Peninsula 20.63%					
Yucatan State 26.59%			Quintana Roo State 16.32%		
Molas 30.61%	Xmatkuil 3.85%	Playa del Carmen 23.81%	Akumal 30.56%	Xcalak 9.45%	Xahuaxol 7.69%

*Leishmania braziliensis *					

Yucatan Peninsula 7.52%					
Yucatan State 11.56%			Quintana Roo State 4.60%		
Molas 10.20%	Xmatkuil 19.23%	Playa del Carmen 3.17%	Akumal 8.33%	Xcalak 4.72%	Xahuaxol 0.00%

*Leishmania infantum *					

Yucatan Peninsula 6.07%					
Yucatan State 1.73%			Quintana Roo State 9.21%		
Molas 2.04%	Xmatkuil 0.00%	Playa del Carmen 3.17%	Akumal 0.00%	Xcalak 15.75%	Xahuaxol 0.00%

**Table 2:** The relationship between positive sera of dogs from the Peninsula of Yucatan (Mexico) and Trypanosomatids, by ELISA and Western blot analysis, against different antigen fractions of *Leishmania braziliensis*.

*Leishmania braziliensis*			

SERA	ELISA		WB
	H	SODe	SODe
1	+		
9		+	+
10		+	+
11		+	+
12		+	+
31		+	+
32		+	−
35		+	+
37		+	+
47		+	+
57		+	+
64		+	+
71		+	+
73		+	+
74		+	−
106		+	+
187		+	+
207		+	+
240		+	+
245	+	+	−
246		+	+
260	+		
262	+		
270		+	−
271		+	+
272	+	+	+
313		+	+
314		+	+
354		+	+
362	+	+	+
363	+	+	+
364		−	+
373		+	+

TOT	9	29	26

^∗^Antigen fraction: Total parasite extract (H) and excreted iron superoxide dismutase by promastigotes from *Leishmania braziliensis* (Fe-SODe).

**Table 3:** The relationship of positive sera of dogs from the Peninsula of Yucatan (Mexico) with Trypanosomatids by ELISA and Western blot analysis against different antigen fractions of *Leishmania infantum*.

*Leishmania infantum*			

SERA	ELISA		WB
	H	SODe	SODe
10		+	+
12		+	+
84	+	+	+
190		+	+
207		+	+
275		+	+
280		+	+
283		+	+
286		+	+
294		+	+
295		+	−
302		+	+
313		+	+
314		+	+
335	+		
358		+	+
360		+	+
361		+	+
362		+	+
363		+	+
364		+	+
365		+	+
370		+	+
372		+	+
373		+	+
374		+	−

TOT	2	25	23

^∗^Antigen fraction: Total parasite extract (H) and excreted iron superoxide dismutase by promastigotes from *Leishmania infantum* (Fe-SODe).

**Table 4:** The relationship of positive sera of dogs from the Peninsula of Yucatan (Mexico) with Trypanosomatids by ELISA and Western blot analysis against different antigen fractions of *Leishmania mexicana*.

*Leishmania mexicana*			

SERA	ELISA		WB
	H	SODe	SODe
9		+	+
11		+	+
12	+	+	+
14		+	+
18		+	+
21		+	+
29		+	+
30		+	+
32		+	+
33		+	+
35		+	+
36		+	+
37		+	+
39	+		
41		+	+
42		+	+
44		+	+
45		+	+
46		+	+
47		+	+
48		+	+
50		+	+
57		+	+
60		+	+
61	+		
71		+	+
72		+	+
73		+	+
83	+	+	+
84	+	+	+
85		+	+
86		+	+
93		+	+
97	+		
98		+	+
102		+	+
104		+	+
106		+	+
108		+	+
109		+	+
110		+	+
115		+	−
123		+	+
125		+	+
130		+	+
133		+	+
134		+	+
137		+	+
142	+		
148	+		
151		+	+
156		+	+
162		+	+
165		+	+
166		+	+
167		+	+
170		+	+
174		+	+
189		+	+
191		+	+
192		+	+
193		+	+
202		+	+
204		+	+
207	+	+	+
220		+	+
221		+	+
223		+	+
224		+	+
225		+	+
231		+	+
232	+		
236	+		
238	+	+	+
239		+	+
240		+	+
245		+	+
246		+	+
260	+		
263		+	+
277		+	+
283		+	+
286		+	+
295		+	+
302		+	+
313		+	+
314		+	+
320		+	+
325		+	+
336	+	+	+
337		+	+
344	+		
393		+	+
406	+	+	+

TOT	16	85	84

^∗^Antigen fraction: Total parasite extract (H) and excreted iron superoxide dismutase by promastigotes from *Leishmania mexicana* (Fe-SODe).

**Table 5:** Detection of co-infection in 412 sera of dogs of the Yucatan Peninsula (Mexico) by ELISA.

Trypanosomatids^a^	No of sera positive	Prevalence
L.mex/L.bra	17	4.13%
L.mex/L.inf	9	2.18%
L.bra/L.inf	8	1.94%
L.mex/L.bra/L.inf	4	0.97%

^a^Promastigote forms of *L. mexicana* (L.mex), *L. braziliensis* (L.bra) and *L. infantum* (L inf).

**Table 6:** Result of the indices that evaluate the reliability of the diagnostic test (ELISA) using the Fe-SODe fraction of the different strains of *Leishmania* in sera of dogs of the Yucatan Peninsula (Mexico).

	*L. braziliensis*	*L. infantum*	*L. mexicana*
Sensitivity	96.20%	100.00%	100.00%
Specificity	99.00%	99.70%	99.50%
Positive predictive value	86.20%	98.80%	92.00%
Negative predictive value	99.70%	100.00%	100.00%
Kappa value	1	1	1
